# Identification of a six‐gene signature with prognostic value for patients with endometrial carcinoma

**DOI:** 10.1002/cam4.1806

**Published:** 2018-10-10

**Authors:** Yizi Wang, Fang Ren, Peng Chen, Shuang Liu, Zixuan Song, Xiaoxin Ma

**Affiliations:** ^1^ Department of Obstetrics and Gynecology Shengjing Hospital of China Medical University Shenyang China

**Keywords:** gene signature, prognosis, rbsurv, The Cancer Genome Atlas, uterine corpus endometrial carcinoma

## Abstract

Uterine corpus endometrial carcinoma (UCEC) is frequently diagnosed among women worldwide. However, there are different prognostic outcomes because of heterogeneity. Thus, the aim of the current study was to identify a gene signature that can predict the prognosis of patients with UCEC. UCEC gene expression profiles were first downloaded from the The Cancer Genome Atlas (TCGA) database. After data processing and forward screening, 11 390 key genes were selected. The UCEC samples were randomly divided into training and testing sets. In total, 996 genes with prognostic value were then examined by univariate Cox survival analysis with a *P*‐value <0.01 in the training set. Next, using robust likelihood‐based survival modeling, we developed a six‐gene signature (*CTSW*,* PCSK4*,* LRRC8D*,* TNFRSF18*,* IHH,* and *CDKN2A*) with a prognostic function in UCEC. A prognostic risk score system was developed by multivariate Cox proportional hazard regression based on this six‐gene signature. According to the Kaplan‐Meier curve, patients in the high‐risk group had significantly poorer overall survival (OS) outcomes than those in the low‐risk group (log‐rank test *P*‐value <0.0001). This signature was further validated in the testing dataset and the entire TCGA dataset. In conclusion, we conducted an integrated study to develop a six‐gene signature for the prognostic prediction of patients with UCEC. Our findings may provide novel biomarkers for prognosis and have significant implications in the understanding of therapeutic targets for UCEC.

## 1 INTRODUCTION

In 2012, uterine cancer among women was frequently diagnosed worldwide.^1^ The mortality rate of uterine corpus endometrial carcinoma (UCEC), one of the most common uterine cancers, is globally increasing.^2^ In China, UCEC is the second most common cancer of the female genital system,^3^ and the 5‐year survival rate is 55.1% in China.^4^ UCEC comprises two major groups: type I, which is hormonally driven and has a good prognosis, and type II, which is hormone independent with a poor prognosis.^5^, ^6^ Therefore, the identification of ways to improve the prognosis or explore significant molecular pathways in UCEC is necessary.

Recently, with the help of microarray and sequencing methods, as well as available open‐access databases such as The Cancer Genome Atlas (TCGA), the identification of molecular subtypes and discovery of biomarkers have been performed in cancers,^7^, ^8^, ^9^ including UCEC.^10^ For example, using RNA‐seq data from the TCGA database, 3742 differentially expressed genes (DEGs) were identified among 552 UCEC samples and 35 normal controls.^10^ A gene co‐expression network was constructed to identify key genes associated with the cell cycle and the tumor protein p53 signaling pathway. However, these studies were only aimed to identify abundant DEGs and biological processes rather than explore key genes related to the prognosis of patients. Two‐hundred and eighteen DEGs have been discovered among survivors (5‐year survival) and nonsurvivors of UCEC using genomewide expression array analysis.^11^ Sun *et al* 12 used the random forest feature selection procedure to establish five long non‐coding RNA (lncRNA) biomarkers for UCEC progression. Moreover, another study detected a novel lncRNA‐focused expression signature including 11 lncRNAs that are associated with the survival of UCEC based on a risk scoring strategy.^13^

Although recent studies have described key biomarkers and gene models for UCEC, a few gene signatures with prognostic value still exist. Additionally, different methods will result in different and various gene signatures, suggesting that robust methods to establish gene models for UCEC are necessary. Moreover, gene expression data can be meaningful in discovering survival‐associated genes for cancer research. However, because of the high‐dimensional difficulty of expression data, sophisticated methods will be necessary. In order to discover survival‐associated genes, methods for survival analysis such as the Cox and the log‐rank test have been applied to various cancer analysis. In view of robustness and convenience, robust likelihood‐based survival modeling was developed. It can select survival‐associated genes which is based on the partial likelihood of the Cox model and separates training and validation sets of samples for robustness.^14^

In this study, we conducted an integrated study to develop a six‐gene signature for the prognostic prediction of patients with UCEC using robust likelihood‐based survival modeling. A prognostic risk scoring system was further established and validated by a testing set and an entire set. To our knowledge, no prior study has focused on the gene signature with prognostic value using the above method in UCEC. Therefore, our findings may provide novel biomarkers for prognosis and have significant implications in the understanding of therapeutic targets for UCEC.

## 2 MATERIALS AND METHODS

### 2.1 Data source and processing

The gene expression profile of UCEC RNA sequencing data fragments per kilobase of exon model per million reads mapped (FPKM) and matched clinicopathological information with follow‐up were downloaded from the TCGA database (https://cancergenome.nih.gov/). FPKM is a normalized estimation of gene expression based on RNA‐seq data. In total, there were 550 UCEC samples selected in our first analysis. The Pearson's correlation coefficient was used to eliminate outlier samples. All patients were then randomly assigned to a training set (n = 228) and a testing set (n = 229). Here, the model is initially built and trained on the training dataset. The testing dataset means a dataset used to provide an unbiased evaluation of a final model fit on the training dataset. The final expression levels of FPKM data were determined by quantile normalization and log2(*x* + 1)‐transformation in the R language environment.

### 2.2 Prognosis‐related gene screening

To screen prognosis‐related genes among all identified genes of UCEC, we performed univariate Cox proportional hazard regression using the survival package in R. Identified genes were considered as statistically significant with a *P*‐value <0.01.

### 2.3 Kyoto Encyclopedia of Genes and Genomes pathway and disease enrichment analysis of the genes

In order to describe the biological roles and functions of the genes, KOBAS (https://kobas.cbi.pku.edu.cn/anno_iden.php) is a web server for functional gene set enrichment.^15^ For the Enrichment module, the gene list was used as the input to generate enriched gene sets, corresponding names, and *P*‐value based on the Kyoto Encyclopedia of Genes and Genomes (KEGG) database. The *ggplot2* R package was used to draw a bubble plot of the enrichment results. All the packages were used in the R language environment.^16^

### 2.4 Gene signature identification

To obtain robust and survival‐associated genes, we constructed a robust likelihood‐based survival model using the *rbsurv* package in R.

1. First, in this model, all 228 UCEC patients were again randomly assigned to a training set with *N *× (1 − *p*) samples and a testing set with *N* × *p* samples (*P* = 1/3). Each identified gene obtained a corresponding parameter and evaluated the log‐likelihood with the parameter estimate and validation dataset.
2. The above procedure was repeated 10 times, resulting in 10 log‐likelihoods for each gene. The best gene with the largest mean log‐likelihood was selected.
3. By evaluating every two‐gene model, we searched the next best gene and selected an optimal one with the largest mean log‐likelihood. A series of models was constructed according to the above procedure.
4. Akaike information criteria (AICs) were computed to select an optimal predictive model with the smallest value.

To analyze differential expression patterns in different pathological stages of UCEC, Gene Expression Profiling Interactive Analysis (GEPIA),^17^ a web server (https://gepia.cancer-pku.cn/index.html) was used to analyze the RNA sequencing expression data from the TCGA database.

### 2.5 Prognostic risk scoring system establishment and validation

To generate a risk scoring system for the six genes, we performed a multivariate Cox proportional hazard regression. First, we obtained the regression coefficient of each gene using the *survival* package in R. The coefficient (the parameter *coef* in R) of each selected gene represented the estimated logarithm of the hazard ratio (HR, the parameter *exp(coef)* in R). Then, a risk score formula was established for all patients. The area under the time‐dependent receiver operating characteristic (ROC) curve (AUC) was determined to predict the 5‐year survival using the *survivalROC* package in R. An optimal cut‐off point was selected as the maximal sensitivity and specificity. According to the optimal cut‐off point, patients were divided into high‐ and low‐risk groups. The Kaplan‐Meier curve was used to assess the survival difference between two groups using the log‐rank test.

To determine the feasibility and reliability of our six‐gene signature, we used the testing set (n = 229) and entire set (n = 457) of TCGA samples. The methods were the same as those described above.

## 3 RESULTS

### 3.1 Data selection and processing of UCEC in TCGA

**Figure 1:**
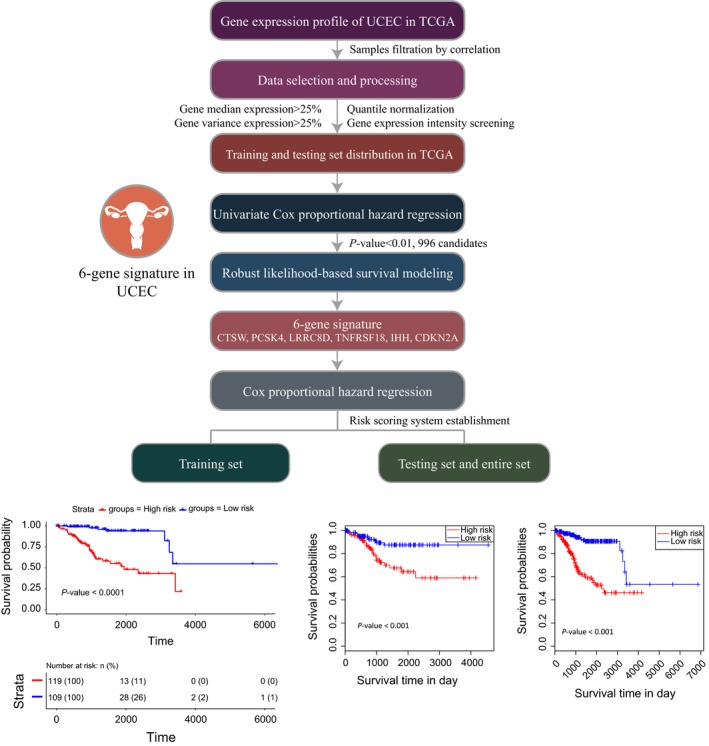
Workflow of the present study to construct a six‐gene signature in UCEC

In the present study, we performed an integrated study to develop a six‐gene signature for the prognostic prediction of patients with UCEC (Figure 1). Based on the gene expression profile in the TCGA database, we first obtained 550 UCEC clinical samples. To eliminate outlier samples, the Pearson's correlation coefficients of these patients were calculated (Figure S1). Combined with the clinical data, we finally selected 457 samples for further analysis. After quantile normalization and log2(*x* + 1)‐transformation, 19 754 gene expression profiles were acquired for these samples from the TGCA database.

To screen key genes with differential expression patterns among UCEC samples, we used the following conditions: (a) The gene expression level was in more than 50% of all samples; (b) The median gene expression level was higher than 25% of all genes; (c) The variance in the gene expression was higher than 25% of all genes. Therefore, we found 11 390 key genes that were abundantly expressed and altered among UCEC samples (Table S1).

### 3.2 Screening of genes with prognostic value in UCEC

To screen genes with prognostic value, we performed survival analyses using a univariate Cox proportional hazard regression model. First, we randomly and averagely divided all TCGA samples into training and testing sets. Univariate survival analysis in the training set identified 996 significantly differentially expressed genes with a *P*‐value <0.01 (Table S2). The top 20 genes significantly associated with survival in the training set are shown in Table 1.

**Table 1:** Top 20 genes significantly associated with survival in the training set by univariate survival analysis

Gene	HR	COX *P*‐value
*GHDC*	0.457557875036391	0.000100021942974871
*FDXR*	0.468925189626395	0.000100716765065179
*WDR18*	0.493466437227939	0.00010197449430549
*TNFRSF18*	0.571784856649668	0.000103100346575125
*NHLRC1*	2.02047364222352	0.000103148076685544
*STX18*	0.646067929363763	0.000104147063068716
*DOHH*	0.403608447444353	0.00010877738870485
*PLEKHM1*	0.268293547262243	0.00011121534741243
*RHBDD3*	0.414219186366586	0.000112316459935924
*SIX1*	1.54774090899324	0.000113214947174201
*ARHGAP29*	1.64562731046508	0.000116204797067754
*SLC25A35*	0.636611361773287	0.00012065727963817
*JPH1*	2.59957982868585	0.000121788237571585
*TSPYL5*	1.45939628562748	0.000128354676291575
*ABHD17A*	0.33113927819869	0.000128826059553888
*ZSWIM7*	0.362493997475613	0.000133382635947754
*KPNA4*	3.05310648179915	0.000134916517875894
*MRPS22*	4.52735643193328	0.000136257618594371
*MED18*	0.324645316660884	0.000139010410348361
*CDKN2A*	1.38840320431511	0.00014262232525053

**Figure 2:**
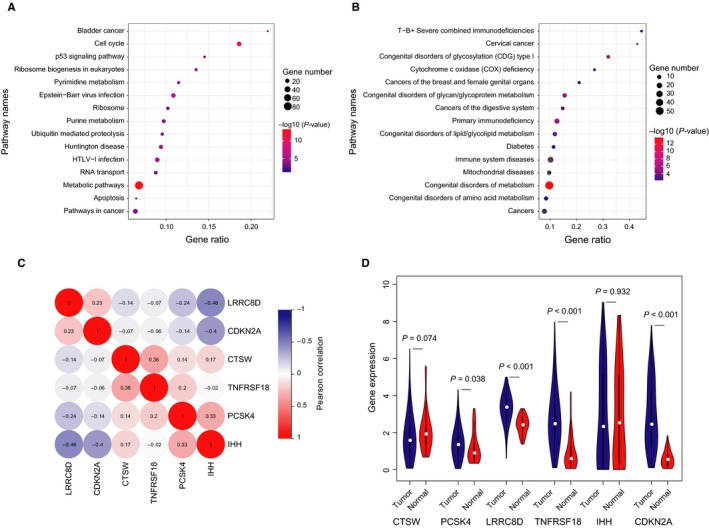
Screening of genes with prognostic value in UCEC. A, Top 15 Kyoto Encyclopedia of Genes and Genomes (KEGG) enrichment pathways based on the KOBAS database. B, Top 15 KEGG enrichment diseases based on the KOBAS database. C, Heatmap of Pearson's correlation coefficient matrix of six genes in the training dataset. D, Violin plots of six‐gene expression profiles in the TCGA database

Using the KOBAS database, KEGG pathway enrichment analysis showed that the above genes were significantly enriched in pathways associated with cell cycle and metabolic pathways. In addition, several pathways related to cancer were also significantly enriched, including the p53 signaling pathway, pathways in cancer and tight junction (Figure 2A, Table S3). KEGG disease enrichment analysis suggested that cancers of the breast and female genital organs were significantly enriched (Figure 2B, Table S3). These results indicated that the above genes play an important role in the pathogenesis and progression of UCEC.

### 3.3 Identification of the six‐gene signature with prognostic value

To build a robust gene model that can predict the prognosis of patients with UCEC, we used robust likelihood‐based survival modeling. Based on this method, we established a six‐gene signature for the prognostic predictor of UCEC. The six prognosis‐related genes selected by AICs were *CTSW*,* PCSK4*,* LRRC8D*,* TNFRSF18*,* IHH,* and *CDKN2A* (Table 2).

**Table 2:** Prognosis‐related six‐gene signature by robust likelihood‐based survival modeling

Gene	nloglik	AIC
*PCSK4*	200.83	403.67^a^
*IHH*	196.83	397.67^a^
*CTSW*	189.64	385.29^a^
*LRRC8D*	188.06	384.12^a^
*TNFRSF18*	184.95	379.91^a^
*CDKN2A*	183.82	379.65^a^
*BATF*	183.8	381.61
*CD3E*	183.53	383.07
*ZDHHC1*	183.4	384.8
*LCK*	182.88	385.76
*CXCR3*	182.24	386.49
*SLC25A35*	182.06	388.11
*ANKRD22*	180.91	387.82
*CD3D*	180.91	389.82
*FDXR*	180.72	391.44
*MRAP2*	180.12	392.23
*TTK*	179.48	392.96
*PLEKHM1*	179.01	394.03
*PPP1R16B*	175.85	389.71

nloglik: negative log‐likelihoods.

a The selected genes by AIC.

**Figure 3:**
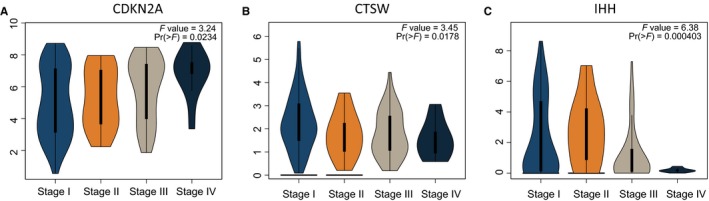
Gene expression patterns between different clinical stages of UCEC. A, Expression patterns of *CDKN2A*. B, Expression patterns of *CTSW*. C, Expression patterns of *IHH*

As shown in Figure 2C, there were weakly positive correlations between *IHH* and *PCSK4*. However, *IHH* and *LRRC8D*,* CDKN2A* presented negative correlations in the training set. When comparing the expression levels of these genes between UCEC and normal tissues, we found that *PCSK4*,* LRRC8D*,* TNFRSF18,* and *CDKN2A* were differentially expressed (Figure 2D). When we explored their expression patterns among different clinical stages of UCEC, we found that *CDKN2A*,* CTSW,* and *IHH* were differentially expressed among different pathological stages using the GEPIA database (Figure 3A‐C). These results suggested that the six‐gene signature may be considered a strong prognostic predictor for patients with UCEC.

### 3.4 Construction of a prognostic risk scoring system using the six‐gene signature

From the above methods, we obtained a six‐gene signature with prognostic value in UCEC. To investigate the association between these six genes and the clinical prognosis of UCEC, we developed a prognostic risk scoring system based on these genes. Using multivariate Cox proportional hazard regression, the survival risk score was calculated as follows: risk score = (−0.4377) × *PCSK4* value + (−0.5322) × *IHH* value +0.4211 × *CTSW* value + (−0.3115) × *LRRC8D* value + (−0.0673) × *TNFRSF18* value + 0.1499 × *CDKN2A* value.

**Figure 4:**
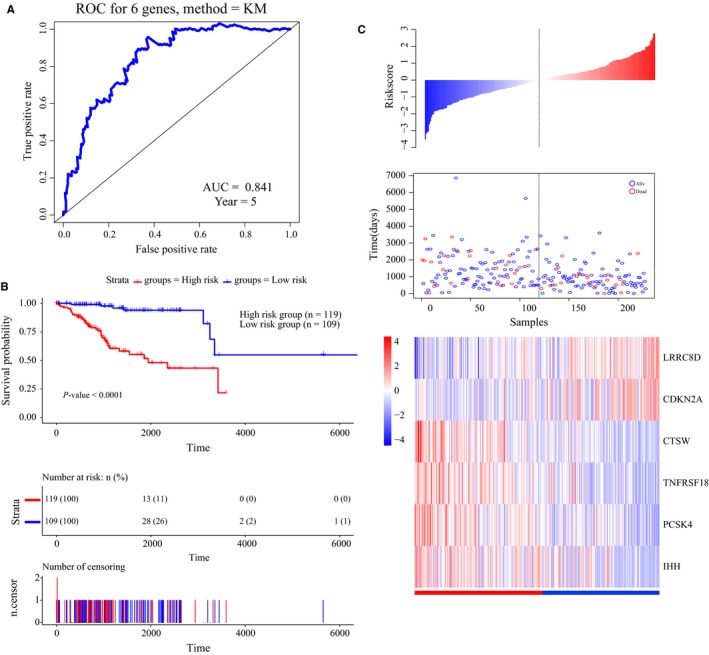
Construction of a prognostic risk scoring system based on the six‐gene signature. A, Time‐dependent receiver operating characteristic (ROC) curve for predicting the 5‐year survival. B, Kaplan‐Meier survival curve of samples divided into high‐ and low‐risk groups according to the optimum cut‐off point (log‐rank test *P*‐value <0.0001). C, Six‐gene signature‐based risk score, patient survival results, and gene expression heatmap

Next, we calculated the risk score for each patient in the training set based on the above results. The concordance index (*C*‐index) was first calculated to evaluate the performance of prediction for this gene signature. The *C*‐index was 0.82 (95%CI: 0.76‐0.88, *P*‐value <0.001). As shown in Figure 4A, the AUC was 0.841, confirming the prediction accuracy of survival prediction based on our six‐gene signature. The patients in the training set were then divided into a high‐risk group (n = 119) and low‐risk group (n = 109) according to the optimum cut‐off point in the ROC curve. The mean and median overall survival (OS) for high‐risk group is 5.80 and 5.33 years, respectively. The mean OS for low‐risk group is 13.96 years. Besides, the 5‐year OS rate for high‐risk group is 55.2% and 94.0% for low‐risk group. From the Kaplan‐Meier curve, patients in the high‐risk group had significantly poorer OS outcomes than those in the low‐risk group (log‐rank test *P*‐value <0.0001) (Figure 4B). The six‐gene signature‐based risk score, patient survival results, and gene expression heatmap are shown in Figure 4C. Therefore, these results indicated the accuracy of the prognostic value based on our six‐gene signature.

### 3.5 Validation of the six‐gene signature in UCEC

**Figure 5:**
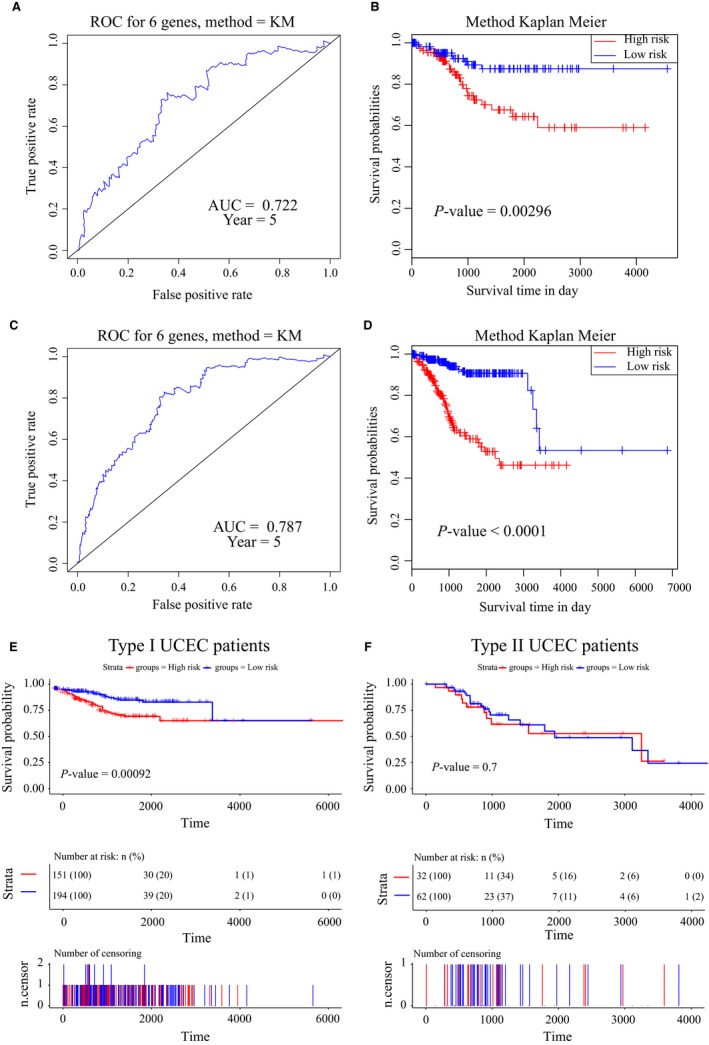
Validation of the six‐gene signature in the validation and entire datasets. A, Time‐dependent receiver operating characteristic (ROC) curve for predicting the 5‐year survival in the validation dataset. B, Kaplan‐Meier survival curve of samples divided into high‐ and low‐risk groups in the validation dataset (log‐rank test *P*‐value = 0.00296). C, Time‐dependent ROC curve for predicting the 5‐year survival in the entire dataset. D, Kaplan‐Meier survival curve of samples divided into high‐ and low‐risk groups in the entire set (log‐rank test *P*‐value <0.0001). E, Kaplan‐Meier survival curve of Type I UCEC samples divided into high‐ and low‐risk groups in the entire set (log‐rank test *P*‐value <0.001). F, Kaplan‐Meier survival curve of Type II UCEC samples divided into high‐ and low‐risk groups in the entire set (log‐rank test *P*‐value >0.05)

To assess the robustness of our six‐gene signature, we validated the signature in the testing dataset and entire TCGA dataset. The survival risk score of each patient in the testing set (n = 229) was calculated based on the above formula. The time‐dependent ROC curve results showed that this six‐gene signature can strongly predict the OS for UCEC patients (AUC = 0.722; Figure 5A). We divided patients into high‐ (n = 104) and low‐risk groups (n = 125) using the optimal cut‐off point. The Kaplan‐Meier curve results showed that there was a significant difference in prognosis between the two groups (log‐rank test *P*‐value = 0.00296; Figure 5B). Moreover, the AUC was 0.787 in the entire TCGA dataset (n = 547, Figure 5C), and the Kaplan‐Meier curves suggested a significantly different survival time in high‐risk patients (n = 211) than in low‐risk patients (n = 246, log‐rank test *P*‐value <0.0001, Figure 5D).

In order to explore whether this six‐gene panel remains predictive roles within Type I UCEC and within Type II UCEC patients, we first divided Type I UCEC patients into high‐ and low‐risk groups. According to the Kaplan‐Meier curve, patients in the high‐risk group had significantly poorer OS outcomes than those in the low‐risk group (log‐rank test *P*‐value <0.001; Figure 5E). Then, the Kaplan‐Meier curve results showed that there was not a significant difference in prognosis between the two groups (log‐rank test *P*‐value >0.05; Figure 5F) in Type II UCEC. These results demonstrated that this six‐gene signature can be used for the prognostic prediction of patients with UCEC. Moreover, this gene signature was also with prognostic value for Type I patients of UCEC. Therefore, this six‐gene signature is robust and strong for the prognostic prediction of patients with UCEC.

## 4 DISCUSSION

We conducted an integrated study to develop a six‐gene signature for the prognostic prediction of patients with UCEC using robust likelihood‐based survival modeling. A prognostic risk scoring system was further established and validated by a testing set and an entire set. Our findings may provide novel biomarkers for prognosis and have significant implications in the understanding of therapeutic targets for UCEC.

Recently, studies have been reported concerning gene signatures for the prognostic prediction in human cancers. All of them developed different gene panels using different methods. For example, using lncRNA expression profiling of 440 clear cell renal cell carcinoma tumors from the TCGA database, a 5‐lncRNA signature was identified to be significantly associated with patient OS.^18^ In univariate Cox regression analysis, five lncRNAs correlated with the patient OS. Next, based on this lncRNA signature, significant differences were found in the survival rate between patients in the high‐ and low‐risk groups. Multivariate Cox regression analysis suggested that the prognostic value of this signature was independent of clinical factors. Another study used a six‐gene signature to predict therapeutic responses in a large panel of cell lines and PDX tumor models of non‐small‐cell lung cancer.^19^ In this study, the methodology and the computational methods gave us great inspiration. The focused concern was allopurinol‐sensitive and allopurinol‐resistant, which has significant clinical application value. Besides, the validation of gene signature was performed in vivo and in vitro.

A robust likelihood‐based survival model is designed to select survival‐associated genes and utilizes a cross‐validation technique that is essential in predictive modeling for data with large variability. Studies based on this method have also been reported for other malignant human tumors. In colorectal cancer, a seven‐gene signature (*NHLRC3*,* ZDHHC21*,* PRR14L*,* CCBL1*,* PTPRB*,* PNPO,* and *PPIP5K2*) was constructed that can predict the OS of patients.^20^ Another study using the Gene Expression Omnibus (GEO) database identified and verified a prognostic nine‐gene expression signature (*NR1I2*,* LGALSL*,* C1ORF198*,* CST2*,* LAMP5*,* FOXS1*,* CES1P1*,* MMP7,* and *COL8A1*) for gastric cancer.^21^ Finally, four genes (*SRPK1*,* PCCA*,* PRLR,* and *FBP1*) with robust prognostic power in the treatment of HER‐2‐negative breast cancer with taxane‐ and anthracycline‐based chemotherapy were identified from the GEO database.^22^ In addition to utilizing gene signatures, several studies have also utilized lncRNA signatures in the model. In lung squamous cell carcinoma, a 4‐lncRNA model was selected with high stability and feasibility. The ideal 4‐lncRNA signature can divide patients with significant prognostic differences.^23^ In gynecological oncology, one study used the TCGA database and this method to build a prognostic 11‐gene model that could function as a prognostic marker in ovarian cancer.^24^ Moreover, a 15‐lncRNA expression signature that can predict cervical cancer patient survival was identified and validated based on the TCGA database using the above method.^25^ However, to our knowledge, no prior study has focused on the gene signature with prognostic value using the above method in UCEC.

We identified six prognosis‐related genes (*CTSW*,* PCSK4*,* LRRC8D*,* TNFRSF18*,* IHH,* and *CDKN2A*) of UCEC in the present study. *TNFRSF18* (tumor necrosis factor receptor superfamily, member 18, also known as *AITR* or *GITR*), a member of the tumor necrosis factor receptor (TNF‐R) superfamily, plays a crucial role in modulating immune response and inflammation.^26^, ^27^, ^28^ It was inactivated during tumor progression in multiple myeloma (MM) through promoter CpG island methylation and was identified as a novel tumor suppressor gene.^29^ Moreover, *GITR* was found to be significantly downregulated in MM patients and cell lines, and its expression can enhance the sensitivity to bortezomib by inhibiting bortezomib‐induced NF‐κB activation.^30^ cyclin‐dependent kinase inhibitor 2A (*CDKN2A*, also known as *ARF*) is frequently mutated or deleted in various tumors and is known to be an important tumor suppressor gene related to different cancer pathways.^31^, ^32^ Interestingly, leucine rich repeat containing eight family (*LRRC8D*, member D) was also shown to be one of six prognosis‐related genes by COX regression analysis in ovarian carcinomas.^33^ The roles of *LRRC8D* in cisplatin and taurine transport were reported.^34^ Additionally, the downregulation of *LRRC8D* expression in ovarian cancer treated with Pt‐drug was found to be associated with reduced survival.^35^ Not only were these three genes involved in cancer pathogenesis, but they were also found to be significant DEGs in our study (*P*‐value <0.001), suggesting that these genes may play important roles in the development and progression of UCEC.

The different genetic alterations found in type I and type II endometrial cancers suggested that these subtypes may have distinct etiologies.^36^ Molecular genetic profile with type I showed defects in DNA‐mismatch repair and mutations in PTEN, K‐ras, and beta‐catenin. Type II showed aneuploidy and p53 mutations. Another study has proved that the expression of beta‐catenin and E‐cadherin in high‐grade endometrial cancers is strongly associated with histological subtype.^37^ Importantly, *CDKN2A* (also known as p16) was helpful in distinguishing serous from endometrioid endometrial carcinomas.^38^ Besides, most of the serous and serous‐like endometrioid tumors exhibited the greatest transcriptional activity of *CDKN2A* in TCGA study.^39^ *CDKN2A* was also found to be upregulated in non‐endometrioid endometrial cancers tissue compared with endometrioid endometrial cancer.^40^ Therefore, these six genes are not trivially classifying Type I vs Type II cancer. This six‐gene signature can also be used for the prognostic prediction of patients with UCEC.

Based on the above results, we performed GO enrichment analysis of these six genes. We found that they were significantly enriched in protein processing, protein metabolic process, peptidase activity, and proteolysis. Moreover, they were enriched in regulation of cell adhesion, regulation of cell death, and regulation of cell proliferation. Therefore, we hypothesize that the mechanism for this six‐gene signature in prognosis of UCEC may be due to the regulation of protein synthesis and metabolism processes as well as biological activities of cells such as cell adhesion, death, and proliferation.

From the results of the Pearson's correlation coefficient among these six genes (Figure 2C), only weak correlations were found. This result suggested that these six genes may exert their molecular functions independently in the pathogenesis of UCEC. Considering the sensitivity and robustness of gene signature, the reproducibility and validation of its association in an independent group of patients were necessary. The prognostic value must demonstrate independence from other standard factors in a multivariate analysis.^41^ These may be common problems for recent studies about gene signature. In our present study, several limitations were identified; for example, this six‐gene signature should be further verified in a large number of clinical samples and other method such as QT‐PCR.

In conclusion, we conducted an integrated study to develop a six‐gene signature for the prognostic prediction of patients with UCEC from the TCGA database. In total, 11 390 key genes were first selected after data processing and forward screening. Additionally, 996 genes with prognostic value were examined by univariate Cox survival analysis, and a six‐gene signature (*CTSW*,* PCSK4*,* LRRC8D*,* TNFRSF18*,* IHH,* and *CDKN2A*) with prognostic function was identified using robust likelihood‐based survival modeling. Patients in the high‐risk group had significantly poorer OS than those in the low‐risk group based on this six‐gene signature.

## CONFLICT OF INTEREST

The authors declare that they have no conflict of interests.

## AUTHORS’ CONTRIBUTIONS

YZW conceived and designed the study. FR and PC contributed to the literature search. YZW, PC, and SL performed this study. YZW and ZXS wrote the initial draft of the manuscript. XXM reviewed and edited the manuscript. All authors read and approved the manuscript.
