# Supplementary figures and images for: Identification of a six‐gene signature with prognostic value for patients with endometrial carcinoma
Source: Cancer Med. 2018 Oct 10;7(11):5632–42. doi: 10.1002/cam4.1806 (PMC6247034; doi:10.1002/cam4.1806)

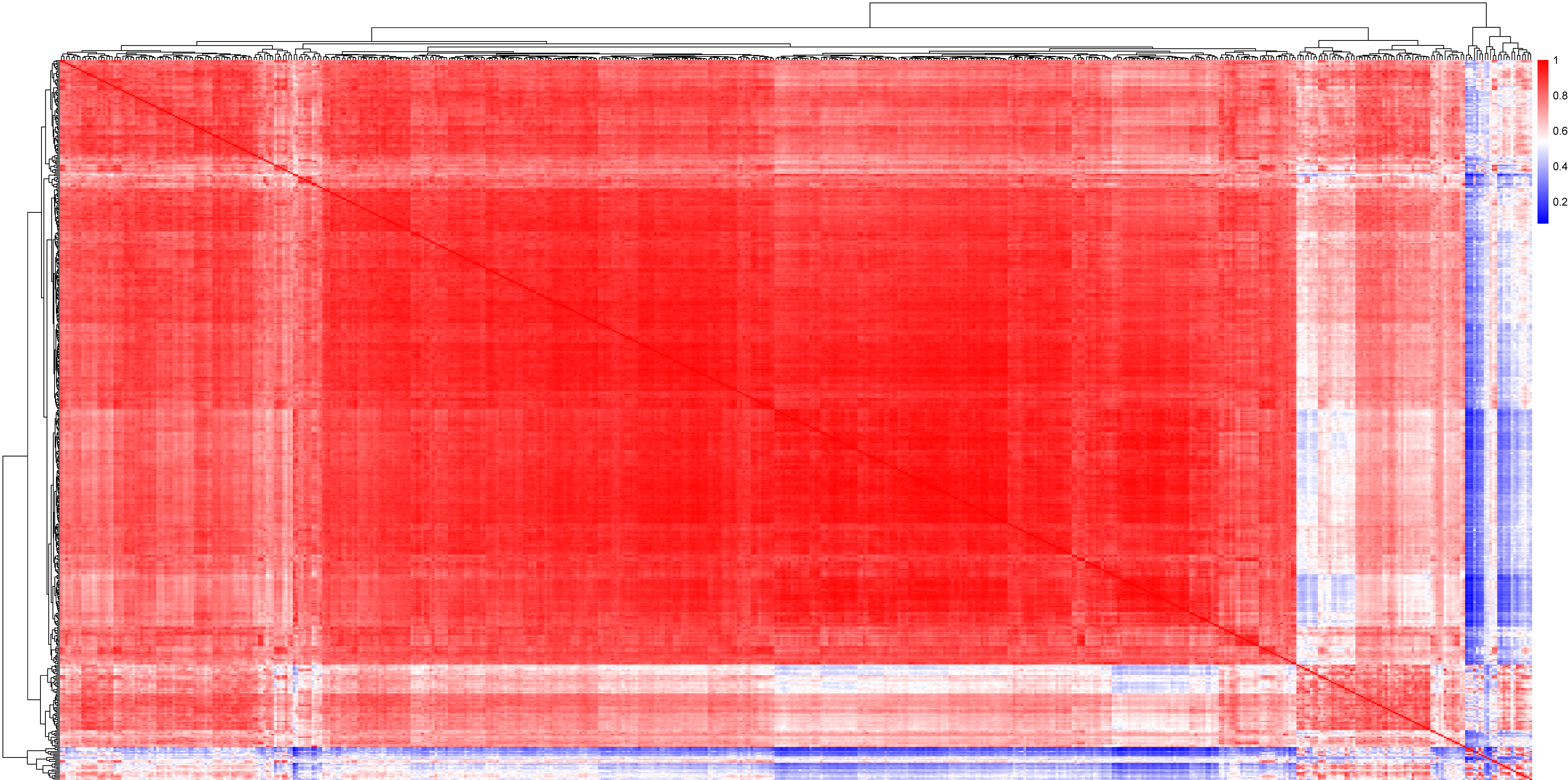

Supplement: Supplementary file 1 [file CAM4-7-5632-s001.tif]
