# Supplementary material for: Identification of a six‐gene signature with prognostic value for patients with endometrial carcinoma
Source: Cancer Med. 2018 Oct 10;7(11):5632–42. doi: 10.1002/cam4.1806 (PMC6247034; doi:10.1002/cam4.1806)
Supplement: Supplementary file 5 [file CAM4-7-5632-s005.docx]

**Supplementary Figure Legends**

**Supplementary Figure 1.** Heatmap of Pearson's correlation coefficient matrix of all UCEC samples in the TCGA dataset.

**Supplementary Table Legends**

**Supplementary Table 1.** Key genes with differential expression patterns among UCEC samples.

**Supplementary Table 2.** Screening of seed genes by univariate survival analysis in the training set.

**Supplementary Table 3.** KEGG pathway and disease enrichment analysis.
